# Preparation of Multicolor Fluorescent Carbon Dots Based on Catechol and *o*-Phthalaldehyde

**DOI:** 10.3390/molecules28145303

**Published:** 2023-07-10

**Authors:** Ming Chen, Fulin Yang, Defa Hou, Yunwu Zheng, Can Liu, Xu Lin, Yan Li, Hao Sun

**Affiliations:** National Joint Engineering Research Center for Highly-Efficient Utilization Technology of Forestry Resources, Southwest Forestry University, Kunming 650224, China; 18987285230@163.com (M.C.); houdefa001@163.com (D.H.); zyw85114@163.com (Y.Z.); liucanswfu@163.com (C.L.); linxunefu@126.com (X.L.); ly97111659@163.com (Y.L.)

**Keywords:** carbon dots, multicolor fluorescent, catechol, *o*-phthalaldehyde, multicolor LED

## Abstract

As the foremost category of carbon materials, carbon dots (CDs) have been extensively applied in many domains because of their special fluorescence features and outstanding biocompatibility. However, in early studies of fluorescent CDs, as the fluorescence wavelength of most CDs was restricted to the blue or green region and was excitation dependent, the application of CDs was limited. In this study, three representative CDs, fluorescing yellow, green, and blue, were synthesized under alkaline, neutral, and acidic circumstances, respectively, while using a hydrothermal method in which catechol and phthalaldehyde acted as carbon sources and methanol functioned as the reaction solvent. The carbon nuclei of the three fluorescent CDs all had comparable graphite structures. The diversity of photoluminescence (PL) emission from these three CDs was attributed mainly to the different sizes of the sp^2^ conjugated structures among them. Mixing synthesized CDs with epoxy resin, three colors (yellow, green, and blue) of LED using CIE coordinates (0.40, 0.44), (0.33, 0.46), and (0.21, 0.22), respectively, were successfully prepared.

## 1. Introduction

Since Xu et al. isolated carbon dots (CDs) with photoluminescence properties from smoke and dust using the arc discharge method in 2004 [1], research on and applications of CDs have been continuously carried out. Researchers have gradually discovered that CDs have exceptional properties, including low toxicity, antibleaching, good biocompatibility [2,3,4], and excellent photostability [5,6,7]. These materials have received considerable attention in the field of nanomaterials, especially in biological imaging [8,9], drug release [10,11], fluorescent probes [12,13], optoelectronic devices [14,15], and other application fields. At present, various methods for synthesizing CDs have been developed, including laser ablation [16], chemical oxidation [17], microwave-assisted techniques [18,19], hydrothermal methods [20,21], electrochemical synthesis [22,23], and solution chemistry [24]. However, in early research on fluorescent CDs, the fluorescence wavelength of most CDs was restricted to the blue or green region and was excitation dependent, which greatly limited the application of these CDs in biological systems [25,26,27]. Therefore, developing a simple strategy to manufacture multicolor emission CDs is desirable.

Many strategies for synthesizing multicolor fluorescent CDs have been proposed. Jiang et al. successfully synthesized three fluorescent CDs with different colors of red, green, and blue using (*o*, *m*, *p*)-phenylenediamine as the precursor of CDs synthesis using a solvothermal method and ethanol as the reaction solvent [28]. This achievement extensively advanced the development of CDs research. On this basis, Lin et al. used *o*-phenylenediamine as a carbon source and DMF as the solvent and prepared green, yellow, and orange fluorescent CDs using a similar solvothermal method [29]. Yang’s group synthesized multicolor fluorescent CDs using (*o*, *m*)-phenylenediamine and citric acid under solar irradiation [30]. Furthermore, researchers have shown through many studies that the conjugated structure of carbon nuclei in CDs can influence their fluorescence properties. However, the above methods are relatively complex, and researchers must simultaneously change two or more conditional factors. As a result, the preparation of fluorescent CDs through single-factor regulation is desirable.

**Figure 1:**
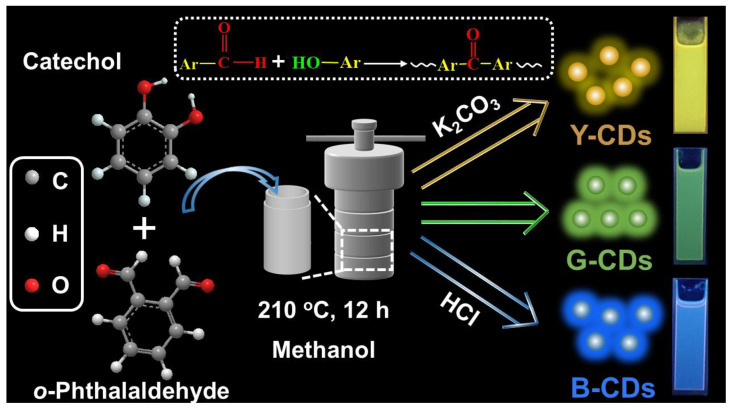
Diagrammatic representation of synthesizing multicolor fluorescent CDs through the simultaneous carbonization of catechol and *o*-phthalaldehyde under different pH levels.

In addition, our group prepared multicolor fluorescent CDs, using tannic acid and *o*-phthalaldehyde as precursors and adjusting pH values, but the synthesis yield of the CDs was relatively low, with a value of only approximately 10% [31]. In this article, we report that at different pH values, catechol and phthalaldehyde reacted to synthesize multicolor fluorescent CDs (as shown in Figure 1). The change of precursor from tannic acid to catechol greatly improved its synthesis yield, reaching approximately 30%. Many phenolic hydroxyl and aldehyde groups in the reaction precursor ensured the high activity of the reaction, and resulted in a larger conjugated system in the synthesis process. The two reaction precursors formed three types of CDs fluorescing yellow, green, and blue under alkaline, neutral, and acidic conditions, respectively. These three CDs were mixed with epoxy resin to obtain polychromatic fluorescent LEDs. Thus, this study offers a new approach for the preparation of multicolor fluorescent CDs and the development of low-cost and environmentally friendly LEDs.

## 2. Results and Discussion

**Figure 2:**
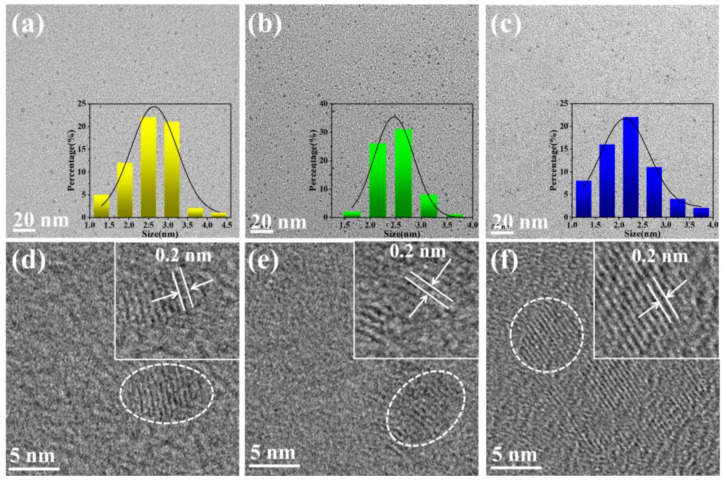
(**a**–**c**) TEM images of Y-CDs, G-CDs, and B-CDs. Insets: particle size distribution. (**d**–**f**) HR-TEM images of Y-CDs, G-CDs, and B-CDs.

The interior morphologies of the prepared CD samples were conspicuously displayed using transmission electron microscopy (TEM). TEM images of the Y-CDs, G-CDs, and B-CDs are shown in Figure 2. Through TEM image analysis of three types of CD samples, we found that the average particle sizes of Y-CDs, G-CDs, and B-CDs were 2.63 ± 0.18 nm, 2.51 ± 0.15 nm, and 2.42 ± 0.11 nm, respectively. Through analysis of high-resolution transmission electron microscopy (HR-TEM) images of Y-CDs, G-CDs, and B-CDs, we found that the lattice spacing of Y-CDs, G-CDs, and B-CDs was not much different; the lattice spacing was 0.2 nm, similar to the (100) planar fringes of graphene [32]. These results indicate that CDs with good crystallization were successfully synthesized using this method. The particle sizes of the three CDs were similar, indicating that the fluorescence mechanism did not mainly arise from the quantum size effect [28,29].

**Figure 3:**
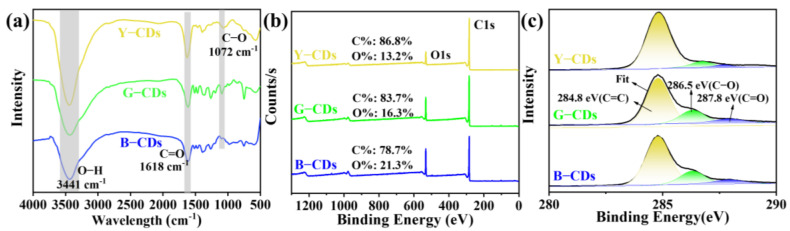
(**a**) FT-IR of Y-CDs, G-CDs, and B-CDs. (**b**) XPS survey, and (**c**) high-resolution C 1s spectra of Y-CDs, G-CDs, and B-CDs in ethanol solution (*c* = 0.1 mg/mL).

Three different types of CDs were characterized using Fourier transform infrared (FT-IR) spectroscopy to explore more details about the functional groups of the surface in the synthesized CDs (Figure 3a). FT-IR analysis of three different CDs samples identified absorption peaks at 3441 cm^−1^, 1618 cm^−1^, and 1072 cm^−1^; the absorption peak at 3441 cm^−1^ corresponds to the stretching vibration of O-H bond. The results showed that the Y-CDs, G-CDs, and B-CDs all had abundant hydrophilic groups, which guaranteed the good solubility of the Y-CDs, G-CDs, and B-CDs in organic solvents [31]. The absorption peak at 1618 cm^−1^ corresponds to the stretching vibration of the C=O bond, and the absorption peak at 1072 cm^−1^ corresponds to the vibration of the C-O bond. Through the above infrared spectrum analysis, we can conclude that these three types of CD samples all had similar chemical bonds and structures.

We utilized X-ray photoelectron spectroscopy (XPS) to analyze the chemical composition of the three types of CDs. The full XPS spectrum clearly showed the ionization of the C 1s (284.7 eV) and O 1s (532.4 eV) peaks (Figure 3b). The decline from 27.0% (B-CDs) to 15.2% (Y-CDs) in the atomic ratio of oxygen to carbon indicates that there was an increasing degree of graphitization in these CDs [32]. Additionally, we observed the peaks of resolved C 1s spectra at 284.8, 286.5, and 287.8 eV in the high-resolution X-ray photoelectron spectra (Figure 3c), which correspond to the existence of C=C/C-C, C-O, and C=O bonds, respectively. Moreover, the gradual decrease in the sp^2^ carbon content from 81.7% (Y-CDs) to 76.2% (B-CDs) indicates that sp^2^ carbon structures were formed in these CDs (Table 1).

**Figure 4:**
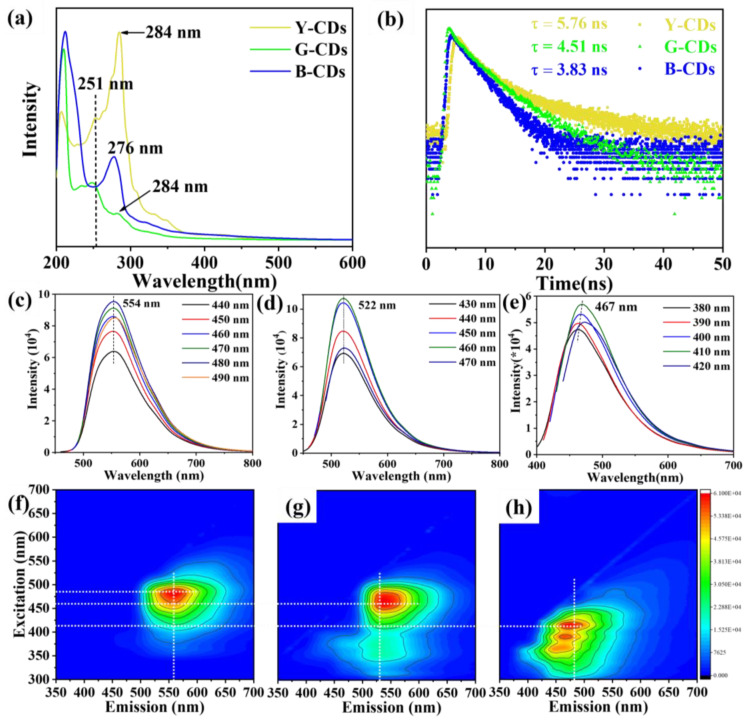
(**a**) UV-Vis absorption spectra of Y-CDs, G-CDs, and B-CDs in ethanol solution (*c* = 0.1 mg/mL). (**b**) Fluorescence lifetime characterization in ethanol solution (*c* = 0.1 mg/mL). (**c**–**e**) Emission, and (**f**–**h**) 3D spectra of Y-CDs, G-CDs, and B-CDs in ethanol solution (*c* = 0.1 mg/mL).

Figure 4a displays the UV-Vis absorption spectra of the three CD samples. In the ultraviolet absorption region, both the Y-CDs and G-CDs exhibit significant absorption peaks at 251 nm and 284 nm. The absorption peak at 251 nm corresponds to the π-π* transition of the C=C bond in the carbon nucleus, while the absorption peak at 284 nm corresponds to the n-π* transition of the C=C bond in the carbon nucleus [13]. In addition, distinct from the UV-Vis absorption spectra of Y-CDs and G-CDs, we observed that there was a characteristic absorption peak at 276 nm in the spectra of B-CDs, which corresponds to the B-absorption peak. These special absorption peaks indicate that the isolated aromatic structure was still preserved in the prepared B-CDs [33]. Distinct from B-CDs, Y-CDs and G-CDs did not give rise to a B-absorption band in their ultraviolet absorption spectra, indicating the presence of longer conjugated structures. Nevertheless, in the low-energy range, these three CD samples did not display any apparent absorption of the surface defect state [33], denoting that the CD fluorescence did not mainly originate from the surface defect state.

Aiming to identify the luminescent center of the prepared CDs, we dissolved three CDs in ethanol and then measured their fluorescence emission (PL) spectra (Figure 4c–e). The maximum emission values of the Y-CDs, G-CDs, and B-CDs were observed to be *λ_max_* = 554 nm, 522 nm, and 467 nm, respectively. Based on the emission results of the three CD samples, a slight reliance on the excitation wavelength was observed in these three CDs [34]. According to the analysis of the fluorescence emission spectra of the three types of CDs, it could be seen from the corresponding fluorescence spectra that the Y-CDs produced a fluorescence peak at 554 nm under an excitation wavelength of 440–490 nm, and had a QY of 24.1% under an excitation wavelength of 460 nm (Figure 4c). For G-CDs, there was significant green emission (Figure 4d). Under an excitation wavelength of 430–470 nm, the emission peak appeared at 522 nm, and the QY was 18.4% under an excitation wavelength of 460 nm. Similarly, for B-CDs, the emission of blue light was apparent (Figure 4e) under excitation wavelengths of 380–420 nm. There is an emission peak at 467 nm and a QY of 16.8% at 380nm excitation. In addition, we also measured the 3D spectra of Y-CDs, G-CDs, and B-CDs (Figure 4f–h); results revealed that each of the three different types of CDs had only one appropriate excitation source, which was consistent with the results of the above fluorescence spectrum analysis. Finally, as shown in Figure 4b, the fluorescence lifetimes of the Y-CDs, G-CDs, and B-CDs were 5.76, 4.51 and 3.83 ns, respectively. Meanwhile, the fluorescence attenuation kinetics of the Y-CDs, G-CDs, and B-CDs were analyzed. It was found that the fluorescence lifetime of the three CD samples decreased gradually with the blueshift of the emission wavelength [30].

Generally, the fluorescence mechanism of CDs is mainly derived from two driving forces; one is primarily caused by various band gap transitions caused by sp^2^ conjugated structures in the carbon nucleus, and the other correlates to the surface defects of CDs [13]. However, there were no surface defects present in the ultraviolet absorption spectra of the CD samples, which convinced us that the mechanism mainly regulating the synthesized CDs fluorescence was the former fluorescence mechanism; the experimental full XPS spectrum clearly showed a decline from 27.0% (B-CDs) to 15.2% (Y-CDs) in the atomic ratio of oxygen to carbon, indicating that there was an increasing degree of graphitization in these CDs. The gradual increase in the sp^2^ carbon content from 76.2% (B-CDs) to 81.7% (Y-CDs) indicates that sp^2^ carbon structures were formed in these CDs. These data confirm the hypothesis of this fluorescence mechanism. The process of synthesis of CDs is greatly impacted by the pH value of the reaction [35,36,37]. Different pH values lead to distinct CDs structures, thereby inducing multicolor luminescence from CDs. To reveal changes in chemical structure over the duration of the carbonization process, we conducted ^1^H and ^13^C nuclear magnetic resonance (NMR) characterization, but the complexity of the spectra and the difficulty of deeper analysis hindered us from conducting a thorough analysis. Acidic conditions discourage the aldehyde group from reacting with the phenolic hydroxyl group of catechol, which presumably contributes to the carbonyl group in *o*-phthalaldehyde being protonated and losing activity, thus leading to a blueshift in the fluorescence. In contrast, alkaline conditions are likely to stimulate aldehyde groups to react with phenolic hydroxyl groups, leading to the formation of larger conjugated structures and fluorescence redshift.

**Figure 5:**
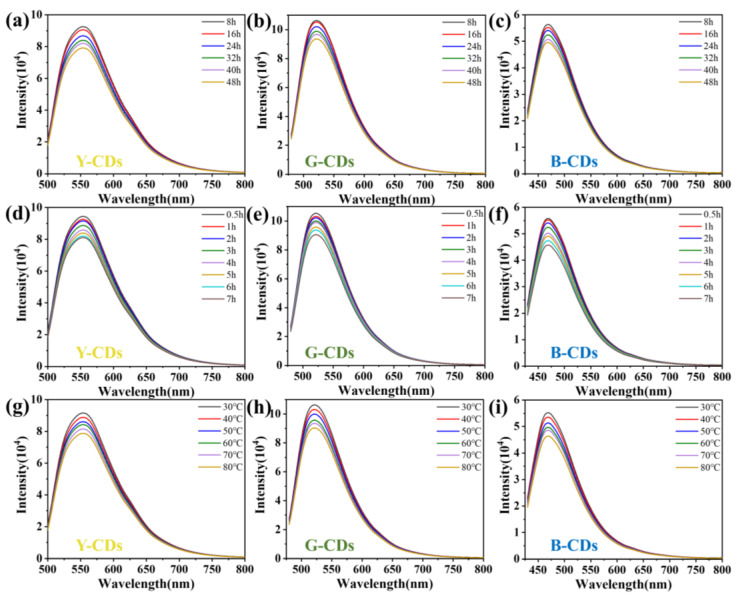
(**a**–**c**) PL emission spectra of Y-CDs, G-CDs, and B-CDs at different durations under visible light in ethanol solution. (**d**–**f**) PL emission spectra of Y-CDs, G-CDs, and B-CDs at different durations under ultraviolet light in ethanol solution. (**g**–**i**) PL emission spectra of Y-CDs, G-CDs, and B-CDs in a water bath at different temperatures in ethanol solution.

**Figure 6:**
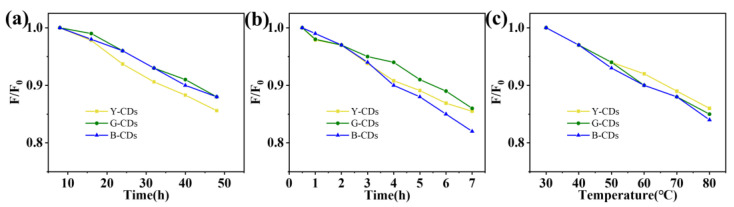
Decay curve of PL intensity of Y-CDs, G-CDs, and B-CDs with increasing (**a**) visible and (**b**) UV irradiation time. (**c**) Decay curve of PL intensity of Y-CDs, G-CDs, and B-CDs with increasing temperature.

**Figure 7:**
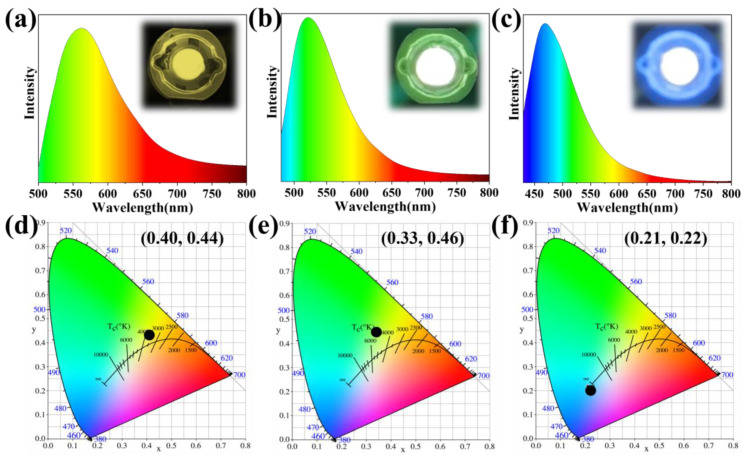
(**a**–**c**) FL emission spectra of Y-LED, G-LED, and B-LED devices. (**d**–**f**) CIE 1931 diagram containing the color coordinates of Y-LED, G-LED, and B-LED devices.

We studied the stability of the three CDs, and all three showed high optical and thermal stability within a maximum decay rate of 20% (Figure 5 and Figure 6); therefore, given that the synthesized Y-CDs, G-CDs, and B-CDs had unique optical properties and excellent color stabilities (Figure 5 and Figure 6), and that the emission peaks in the fluorescence spectra all had a certain peak width [38], these compounds can be used to fabricate multicolor LED lighting materials (Figure 7a–c). The three types of CDs were thoroughly mixed with epoxy resin and then deposited and coated on a chip with a luminescent center of 365 nm. They were then heated in an 80 °C oven for 4 h, and polychromatic LEDs prepared from Y-CDs, G-CDs, and B-CDs were obtained. The CIE coordinates of the Y-CDs, G-CDs, and B-CDs were determined using the LED spectra to be (0.4, 0.44), (0.33, 0.46), and (0.21, 0.22), respectively (Figure 7d–f). The fluorescence emission peak positions of Y-CDs, G-CDs, and B-CDs in the three LED spectra were partially shifted, which was highly likely due to the distinct polarity of the solvent and epoxy resin, resulting in the shift of their emission peaks [39,40].

## 3. Materials and Methods

### 3.1. Materials

*o*-phthalaldehyde (98.0%), catechol (98.0%), methanol (99.5%), ethanol (99.7%), hydrochloric acid (HCl), and potassium carbonate (K_2_CO_3_) were all obtained from Shanghai Titan Science. All reagents applied did not require additional purification unless otherwise stated.

### 3.2. Instruments and Methodology

The conspicuous pictures of transmission electron microscopy (TEM) were captured utilizing an FEI Tecani G2 F20 operating at an acceleration voltage of 200 kV. A Shimadzu UV-2600 spectrometer was used to record the CDs’ UV-Vis spectra. Measurements of fluorescence were conducted using a Shimadzu RF-6000 fluorescence spectrophotometer. Via the KBr pellet method, the Fourier transform infrared (FT-IR) spectrum was acquired under transmission mode using a Thermal Scientific Nicolet iS5 spectrometer. X-ray photoelectron spectroscopy (XPS) was obtained using an Al K excitation K-Alpha spectrometer with a single X-ray source. Binding energy calibration was performed based on C 1s at 284.7 eV. A 290 nm (<1 ns) and a 485 nm (200 ps) nano-LED light source was used to excite the samples. The CIE chromaticity coordinates were measured using a KONICA MINOLTA CS-150 colorimeter. Absolute quantum yield (QY) measurements were performed using a FLS1000 spectrometer (Techcomp, Livingston, Edinburgh, UK) equipped with a calibrated integrating sphere. As the QY was determined by the ratio between photons emitted and absorbed by CDs, it was desirable to place the ethanol solution of CDs in a cuvette to measure their QY, while using solvent ethanol as a blank sample for the reference measurement.

### 3.3. Synthesis of Multicolour CDs

In order to obtain the green suspension, catechol (0.55 g) was mixed with catecaldehyde (0.65 g) in 10 mL methanol solvent, and the mixture was transferred to an autoclave surrounded by a polytetrafluoroethylene wall, which was then placed in a muffle furnace and heated at 210 °C for 12 h. At the same time, using the same operation steps and the same reaction precursor and solvent, distinct from making the green suspension, 0.1 g potassium carbonate (K_2_CO_3_) and 0.5 mL hydrochloric acid (HCl) were each added to one of two autoclaves to change the pH value of the reaction conditions, and finally, we successfully synthesized the yellow and blue suspensions, respectively. Before refining three types of suspensions containing CDs with silica gel, it was imperative to filter the suspensions in order to remove the impurities. After refining three types of suspensions, the crude products were obtained, and then the methylene chloride and ethanol were combined as eluent to eluate three types of crude products. The above procedures were repeated several times to remove excess reactants and reaction precursors from the suspensions; finally, the yellow (Y)-CD, green (G)-CD and blue (B)-CD products were produced. The yields of CDs were 31.2 ± 1.7%, 30.1 ± 1.6%, and 28.5 ± 1.8% for Y-CDs, G-CDs, and B-CDs, respectively.

### 3.4. Preparation of CDs-LEDs

A 1.0 mg carbon dot was thoroughly combined with 5.0 mL of epoxy resin before being cast onto a light-emitting diode (LED) chip which fluoresced purple. The mixture was horizontal and smooth on a non-bubble-doped chip’s surface. In order to make an LED device, an ultraviolet chip with a 365 nm emission wavelength was mounted to an LED base.

## 4. Conclusions

In conclusion, catechol and *o*-phthalaldehyde can be used as precursors in a straightforward and practical solvothermal method to prepare Y-CDs, G-CDs, and B-CDs. Under neutral and alkaline conditions, the two precursor reactions generate green and yellow fluorescent carbon dots, respectively, while under acidic conditions, they react to generate blue fluorescent CDs. The high reactivity of phenolic and aldehyde groups can expand the conjugated structure of CDs and ultimately cause the emission wavelength of CDs to redshift. Moreover, it was discovered that choosing reaction conditions (such as alkaline, neutral, and acidic) is crucial, as reaction conditions regulate the dehydration and subsequent carbonization of precursors, which affect the size of the sp^2^-conjugated domain and produce varied luminescence colors. Our synthesized CDs can be mixed with epoxy resin to produce various colors of LEDs, including Y-LED (CIE: 0.40, 0.44), G-LED (CIE: 0.33, 0.46), and B-LED (CIE: 0.21, 0.22). This study provides a new method for preparing polychromatic fluorescent CDs and developing low-cost and environmentally friendly LEDs.

## Figures and Tables

**Table 1:** XPS spectra data of Y-CDs, G-CDs and B-CDs.

		Y-CDs (%)	G-CDs (%)	B-CDs (%)
XPS survey	C1s	86.8	83.7	78.7
	O1s	13.2	16.3	21.3
C 1s	C=C/C–C	81.7	77.2	76.2
	C–O	14.7	20.1	20.3
	C=O	3.6	2.7	3.5

## Data Availability

Data presented in this study are available on request from the corresponding author.
